# Hydrophobic Modification of Cellulose Acetate and Its Application in the Field of Water Treatment: A Review

**DOI:** 10.3390/molecules29215127

**Published:** 2024-10-30

**Authors:** Yaxin An, Fu Li, Youbo Di, Xiangbing Zhang, Jianjun Lu, Le Wang, Zhifeng Yan, Wei Wang, Mei Liu, Pengfei Fei

**Affiliations:** 1 College of Textile Engineering, Taiyuan University of Technology, Jinzhong 030600, China; anyaxin0046@163.com (Y.A.); lifu@tyut.edu.cn (F.L.); diyoubo@tyut.edu.cn (Y.D.); lujianjun@tyut.edu.cn (J.L.); wangle9106@163.com (L.W.); yanzhifeng0234@163.com (Z.Y.); wangwei03@tyut.edu.cn (W.W.); 2 Shanxi Greenland Textile Co., Ltd., Jincheng 048100, China; 13834310902@163.com; 3 College of Textiles and Apparel, Quanzhou Normal University, Quanzhou 362000, China

**Keywords:** cellulose acetate, membranes, hydrophobic modification, separation, filtration, water purification

## Abstract

With the inherent demand for hydrophobic materials in processes such as membrane distillation and unidirectional moisture conduction, the preparation and application development of profiles such as modified cellulose acetate membranes that have both hydrophobic functions and biological properties have become a research hotspot. Compared with the petrochemical polymer materials used in conventional hydrophobic membrane preparation, cellulose acetate, as the most important cellulose derivative, exhibits many advantages, such as a high natural abundance, good film forming, and easy modification and biodegradability, and it is a promising polymer raw material for environmental purification. This paper focuses on the research progress of the hydrophobic cellulose acetate preparation process and its current application in the water-treatment and resource-utilization fields. It provides a detailed introduction and comparison of the technical characteristics, existing problems, and development trends of micro- and nanostructure and chemical functional surface construction in the hydrophobic modification of cellulose acetate. Further review was conducted and elaborated on the applications of hydrophobic cellulose acetate membranes and other profiles in oil–water separation, brine desalination, water-repellent protective materials, and other separation/filtration fields. Based on the analysis of the technological and performance advantages of profile products such as hydrophobic cellulose acetate membranes, it is noted that key issues need to be addressed and urgently resolved for the further development of hydrophobic cellulose acetate membranes. This will provide a reference basis for the expansion and application of high-performance cellulose acetate membrane products in the environmental field.

## 1. Introduction

While developing rapidly, industries represented by chemicals, textiles, and pharmaceuticals have caused a series of environmental problems due to the discharge of industrial wastewater and pollution [1,2]. At the same time, along with the rapid population growth worldwide, the shortage of freshwater resources has become a key constraint to the survival and development of human society [3,4]. It is expected that by 2030, two-thirds of the world’s population will reside in freshwater-scarce countries, which brings great challenges to water-resource utilization and management [5,6]. Therefore, how to efficiently purify highly polluted wastewater and desalinate seawater, along with the resourceful recycling of complex water systems, has become a worldwide research hotspot in academics and engineering [7,8].

In recent years, membrane-separation technology has been widely used in the fields of oil–water separation, desalination of seawater and brackish water, and other disciplines, and it reflects the characteristics of simple operation, low cost, low energy consumption, and environmental friendliness [9,10,11,12]. Compared with the petrochemical polymer polyvinylidene fluoride (PVDF), polytetrafluoroethylene (PTFE), and other materials that exist within a lack of technology and serious pollution of the essence of the problem, it is of strategic significance to adopt appropriate preparation and separation processes for renewable resources to achieve the efficient recovery of target water resources as an important direction for alleviating the shortage of freshwater resources in China and represents a key to the construction of an eco-friendly society [13,14]. Cellulose is the most widely used and abundant natural material in nature [15,16]. However, its insolubility, strong hydrogen bonding, and high hydrophilicity greatly limit its application [17,18,19,20]; thus, researchers have turned their attention to cellulose derivatives, i.e., cellulose acetate (CA) and its modified polymers.

CA was one of the earliest materials used to manufacture filtration membranes, and it is processed into different types of membrane components for ultrafiltration, nanofiltration, reverse osmosis, and other forms of membrane-separation processes. It plays an important role in fields such as biotechnology, the food industry, pharmaceuticals, and the medical industry [21]. In recent years, research on the separation of membranes has gradually expanded from traditional fields to new areas such as membrane distillation, hydrophobic permeability, and unidirectional moisture conductivity. Among these fields, the film-forming characteristics and biomass properties of CA make it a favored polymer raw material for new-demand membrane products. However, it should be noted that CA belongs to the category of hydrophilic polymers [22], which limits its application in hydrophobic membranes such as membrane distillation and hydrophobic permeability. Therefore, the targeted hydrophobic modification of CA or its membrane products is crucial for improving membrane performance and expanding membrane applications [23], and it is also a key prerequisite and effective strategy for preparing new high-performance CA separation membranes. Accordingly, this paper summarizes the commonly used methods for CA hydrophobic modification from two aspects: the construction of hydrophobic surfaces with special microstructures and the chemical functional properties. Further, the separation and purification functions of hydrophobic CA profiles in the field of environmental purification are emphasized, with the aim of providing a methodological reference and theoretical basis for the research and development of CA hydrophobic membranes.

## 2. Hydrophobic Modification of Cellulose Acetate

### 2.1. Hydrophobicity Mechanism

**Figure 1:**
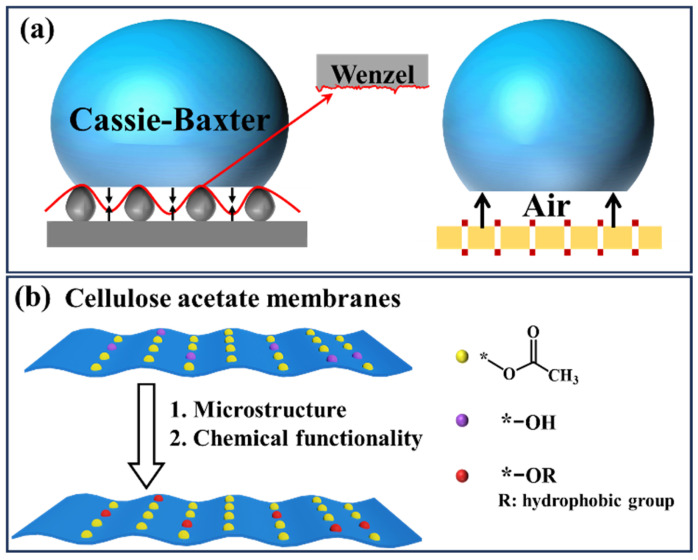
Schematic diagram of hydrophobic interaction mechanism: (**a**) surface microstructure; (**b**) hydrophobic groups/substances.

The hydrophobic function of membrane materials and other profiles is achieved by constructing hydrophobic surfaces. The construction of hydrophobic surfaces can be achieved by both surface microstructural design and surface chemical functional regulation. Surface microstructural design refers to changing the microstructure of the material’s surface through morphological design (e.g., co-blending, deposition/coating, or chemical etching) to form gaps that can enhance the energy barrier for the liquid to penetrate the membrane material, thus obtaining a hydrophobic effect. Microstructure design can be achieved in situ or by modifying the membrane surface through post-treatment processes. The hydrophobic mechanism can be analyzed through the Cassie–Wenzel theoretical model (as shown in Figure 1a): unlike the close contact between (water) droplets and smooth surfaces, when liquid droplets come into contact with rough membrane surfaces, air molecules existing between the rough particle structures form micro air pockets. This process hinders the contact between the solid surface and the liquid and reduces liquid droplet infiltration, thereby producing hydrophobic effects [24]. Within a certain range, the increase of membrane surface roughness can help to form larger-volume air pockets, creating a synergistic effect of hydrostatic pressure, capillary force, and surface hydrophobicity, further enhancing hydrophobic performance.

Unlike the surface-roughness microstructure design, chemical functionality is achieved by modifying the surface of the material through blending/coating or chemical grafting/cross-linking, introducing chemicals with hydrophobic groups or low surface energy (aliphatic chains, aromatic rings, organofluorines, organosilicons, etc.) to achieve hydrophobicity. Both of these strategies can also be organically coupled for the construction of hydrophobic surfaces [25,26,27,28]. Figure 1b shows the mode of action of hydrophobic groups/substances, which may be due to the weak polarity and weak (or even non-existent) hydrogen bonding properties of hydrophobic groups, resulting in a weak affinity with liquid water molecules and macroscopic hydrophobic properties. It should be noted that the effects of microstructure and chemical functionality on macroscopic hydrophobic properties are not isolated. The hydrophobic modification of profiles such as separation membranes is usually achieved through the synergistic coupling of both microstructure and chemical functionality.

### 2.2. Microstructural Hydrophobic Modification

#### 2.2.1. In Situ Construction of Hydrophobic Surfaces

The in situ construction of membrane surfaces with rough structures is achieved by improving the molding process or adding exogenous substances (e.g., organic compounds, inorganic particles) or by combining the two methods.

(1) Improved molding process: Zhang et al. [29] added a thermally induced process to the traditional non solvent-induced phase separation (NIPS) film-forming process, i.e., the CA profiles with a unique nano-micron pore structure were prepared by the thermally induced-non solvent-induced phase separation (TINIPS) technique. In the process of polymer and solvent phase conversion, thermal induction technology can refine the porous structure and promote the formation of interconnected pores in the solvent phase, which not only increases the surface roughness of the material but also facilitates the capture of air and reduces the water contact area. The results showed that the profiles had a wide pore size distribution (2–136 nm), a large specific surface area (54.45 m^2^·g^−1^), and a high porosity (92.1%), and they exhibited excellent hydrophobic properties, with a water contact angle (WCA) of 147°. Liu et al. [30] quenched cellulose triacetate (CTA) with a mass fraction of 5% (180 min, −20 °C) and then obtained a “bead string” structure nanofiber membrane with a diameter of 110 ± 2.8 nm through thermally induced phase separation (TIPS). The appropriate quenching time was beneficial for forming a uniform fiber film. The suitable temperature was conducive to the process of crystal nucleation and growth. When the solution concentration was too low, the phase separation was mainly based on the spinodal liquid–liquid phase separation, which resulted in the forming of fibrous structures. When the solution concentration was too high, nucleation and growth dominated the phase separation, which can easily form particle- or sheet-like structures. The large specific surface area and high porosity made the surface of the nanofiber membrane rougher, and the WCA increased by 59% compared to the cast film, up to 137.5°. Wang et al. [31] prepared CA-polyacrylonitrile (PAN) composite fiber membranes using a multi-channel electrospinning process. By adjusting the mass ratio of CA and PAN, the morphology of the membrane changed from smooth to rough on the nanoscale, with an average diameter increasing from 303 nm to 502 nm and the hydrophobicity increasing (the WCA increased from 86° to 131°). Yoon et al. [32] controlled the diameter of nanofibers by modulating the ratio of dichloromethane and ethanol in the solvent. Finer fibers helped to construct macroscopic roughness between the fibers up to a WCA of 141.6 ± 2.2° and a water tilt angle (WTA) of 12.1 ± 1.4°. Kakunuri et al. [33,34] prepared drug-loaded CA nanofibers by using non-conducting nylon mesh as a template. After peeling off from the template, the side toward the nylon mesh showed hydrophobicity (WCA = 130°–140°), while the other side still showed hydrophilicity (WCA = 30°). This was because the air was captured in the disconnected air gap, so the patterned nanofiber mats showed hydrophobic behavior generated by non-wetting capillary pressure. However, stretching behavior could cause a certain irreversible decrease in hydrophobicity.

The improvement of the molding process can encourage the CA membrane and other profiles to complete the directional construction of a special roughness structure. In turn, it promotes the formation of more durable and stable hydrophobic properties in products. This is an effective strategy for achieving the transformation of CA intrinsic characteristics. However, there are some limitations in this type of method: Firstly, the improvement strategy of the molding process usually requires the introduction of new processes or new technologies, which increases the complexity of the film-formation process. Secondly, while promoting the formation of micro-rough structures, new processes or technologies must comprehensively consider the application characteristics (e.g., membrane-separation efficiency) and other comprehensive properties of raw materials and profile products, resulting in limited process choices. Thirdly, new processes or technologies often have uniqueness or specificity, which greatly limits the universality and promotion of the strategy.

(2) Introduction of exogenous substances: Lim and Kim [35] proposed a geometric structure design with high liquid resistance/repulsion using the Cassie–Baxter model calculations and prepared bead-like nanofibers by adding SiO_2_ particles to the spinning solution. The addition of SiO_2_ formed spherical or bell-shaped protrusions on the nanofibers’ surface, increasing the surface roughness of the fibers and increasing the WCA from 91° to 108°. Anita et al. [36] prepared CA nanofiber membranes coated with zinc oxide (ZnO) nanoparticles. The increase in surface roughness and the presence of ZnO enabled the material to have better water-repellent properties (WCA = 124°) than pure CA membranes (WCA = 47°). Nachev et al. [37] prepared CA-CA/ZnO nanofiber membranes using the pair spray method, achieving superhydrophobicity (152°), while ZnO also endowed the material with certain antifungal properties. Wang et al. [38] added graphene to the CA solution to prepare membrane materials by TINIPS technology. The addition of graphene transformed the microstructure of the material from fiber bundles to a porous structure with particle size. This not only widened the pore size but also increased the surface roughness, with a WCA of 151°.

Introducing exogenous substances to increase the surface roughness of profiles is an effective measure to improve the hydrophobic properties of CA profiles. Inorganic particles are co-mixed in the precursor solution system, such as the casting solution (or spinning solution), and the uniform dispersion of particles on the surface during the forming process is used to construct a hydrophobic layer. This technology has the advantages of a wide material selection range, low technical barriers, and diversified functions, and it has great prospects in macroscopic preparation. However, the aggregation of nano-micron inorganic particles in the precursor solution system, the uneven dispersion of particle components at the interface, and the leakage of exogenous additives during application are the key issues that constrain the hydrophobic stability and application continuity of membrane products. These are also technical difficulties that urgently need to be overcome by this strategy.

#### 2.2.2. Post-Treatment for Constructing Hydrophobic Surfaces

**Figure 2:**
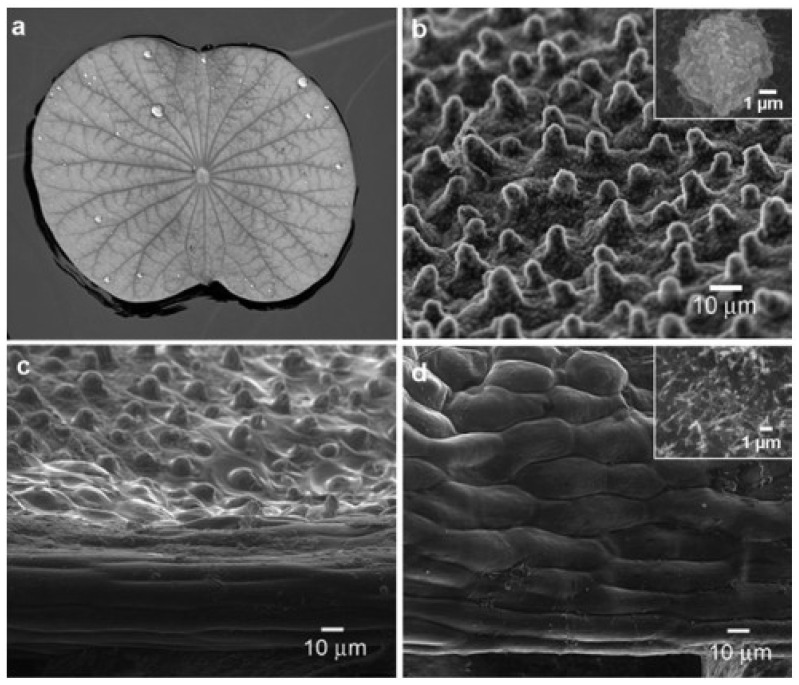
(**a**) Photo of lotus leaf on water; (**b**) ESEM image of lotus leaf surface; illustration is enlarged image of the papilla structure; (**c**) ESEM image of lotus leaf edge; (**d**) ESEM image of lotus leaf edge; illustration is enlarged image of nano-scale wax crystals [42].

The team led by academician Jiang Lei from the Chinese Academy of Sciences discovered that the superhydrophobic properties of lotus leaves are due to the specific nanometer-scale particle structure on their surface (Figure 2), laying a theoretical and research foundation for obtaining CA hydrophobic membranes based on rough structure. The physical or chemical processing of the inherent surfaces (or interfaces) of separation membranes and other profiles to change the surface geometry or surface energy is an important means of improving the surface hydrophobicity/repulsion properties. With the help of the positive electrolyte properties of poly(diallyldimethylammonium chloride), Zhou et al. [39] deposited SiO_2_ nanoparticles onto the CA membranes through electrostatic interactions, increasing surface roughness and offsetting the increase in hydrophilicity due to the addition of chitosan. The WCA reached 145 ± 0.08°. Tasleem et al. [40] successfully synthesized monodisperse linear transparent SiO_2_ with the sol–gel method using a chemical surfactant (trimethoxyoctylsilane (C_11_H_26_O_3_Si)) and a green surfactant (lotus root/lotus leaf extract) and coated it on cellulose acetate filter paper using a vacuum filtration method. Due to the large specific surface area and high roughness of self-assembled silica, its porous structure can form a superhydrophobic layer (WCA = 154°) with micro air pockets. When the post-treatment modification is conducted using physical methods, it is susceptible to external forces leading to the displacement of micro/nanoparticles or separation from the membrane surface, and the sustainability of the modification effect needs to be further verified. However, this method makes it possible to prepare a series of environmental purification membranes with different retention rates. Xu et al. [41] prepared CTA thin films with ordered honeycomb structures with a WCA of 100 ± 1.0° using the respiration chart method. To further obtain heterogeneous morphology, the honeycomb structure was simply torn off from the top layer to become a needle-like structure with a WCA of up to 120 ± 1.3°.

The modulation and improvement of the surface hydrophobicity of CA membranes by post-processing to construct surface microstructures is a highly promising and effective strategy, and this technique makes it possible to prepare environmental purification membranes with different retention rates. However, the developmental stage of surface microstructure construction needs to focus on the uniformity and stability of the binding of micro/nanoparticles to ensure the sustainability and durability of the modification effect. On the one hand, the construction of micro-rough hydrophobic surfaces by post-processing needs to balance the separation membrane layer’s structural integrity and the uniformity of the rough structure. On the other hand, the micro/nanoparticles used to construct the rough structure require long-term stability to avoid detachment.

### 2.3. Chemical Functional Hydrophobicity Modification

#### 2.3.1. Organic Silicon/Fluorine Modifiers

**Figure 3:**
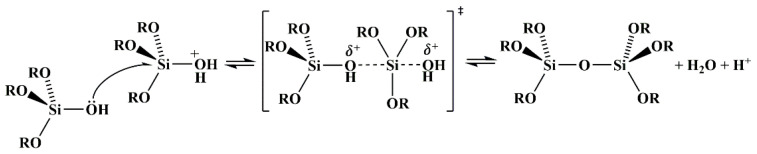
Acid-catalyzed condensation reactions of silanol salts [45].

**Figure 4:**
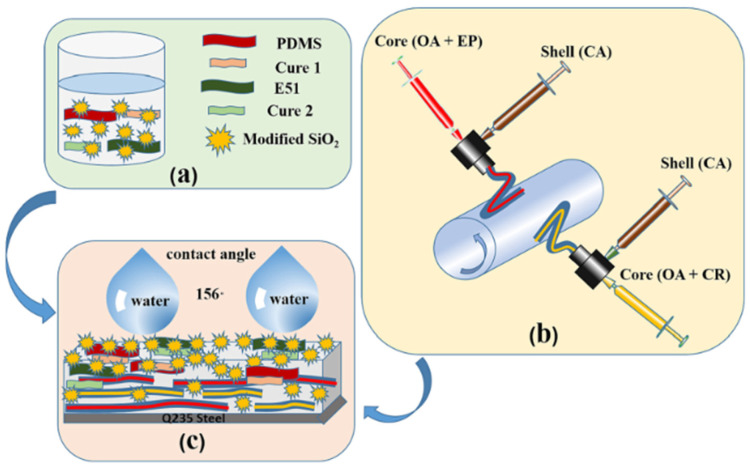
Schematic of the method used to prepare superhydrophobic self-healing CA@(OA + EP1)@(PDMS + EP2)@SiO_2_ core–shell fiber/epoxy coatings [46]: (**a**) preparation of superhydrophobic modified SiO_2_ powder; (**b**) preparation of core-shell fibers/epoxy coating; (**c**) schematic diagram of hydrophobic mechanism.

Organosilicon/fluorine polymers are the most commonly used hydrophobic modifiers for fibers and textiles. Stiubianu et al. [43] used Pt-catalyzed dehydrocoupling to cross-link CA with 1,1,3,3-tetramethyldisiloxane, resulting in a decrease in the wettability of the membrane (an increase of 10.8% in the forward angle and 32.5% in the backward angle), but overall it remained within the hydrophilic range. Afterward, the team [44] chemically modified CA through allylation and cross-linking with siloxane derivatives. With the introduction of siloxane, the surface properties of the cross-linked fiber membrane changed, and the WCA reached over 100°. Danks et al. [45] formed a hydrophobic organic silane layer on the membrane surface through a condensation reaction between the silane coupling agent and hydroxyl groups on the CA surface under acidic conditions (Figure 3). Ji et al. [46] encapsulated oleic acid and epoxy resin with CA to form core–shell nanofibers, and they then coated the surface with an epoxy coating containing polydimethylsiloxane (PDMS)/SiO_2_ to achieve multiple properties, including superhydrophobicity (157.3 ± 3°), excellent wear resistance, high chemical stability, and self-repair (Figure 4). Among those properties, the hydrophobicity was mainly due to the high migration trend of PDMS toward the surface of the epoxy coatings, which generated Si-CH_3_ groups and reduced the surface energy of the coating. SiO_2_ nanoparticles can increase surface roughness and improve the mechanical strength and water contact angle of polymer coatings.

Fluorinated polymers, especially perfluorinated chain polymers (−C_n_F_2n+1_, *n* ≥ 8), exhibit excellent water and oil resistance due to their lower surface energy, and they have received extensive attention in hydrophobic modification. Ding et al. [47] modified CA/SiO_2_ composite nanofiber membranes with perfluoroalkylsilane (FAS)/n-hexane dispersion. The FAS coating layer acted as a “waxy” substance similar to the surface of lotus leaves, reducing the surface energy of the composite membrane. The WCA could reach 156°, demonstrating strong anti-wetting properties. Zhang et al. [48] used perfluorotriethoxysilane (PFDS) as a hydrophobic agent to react with the dehydrogenated hydroxyl groups in the cellulose acetate aerogels (CAAs) molecular chain in a chemical vapor-phase, which connected the long fluorine-containing chains to the main chains via the formation of chemical covalent bonds. The hydrophobic treatment reduced the hydroxyl groups of CAAs and introduced low surface energy PFDS, resulting in better hydrophobicity and an increase in the WCA from 55° to 136°. Xu et al. [41] further modified CTA films with honeycomb and needle-like structures using perfluorotrichlorosilane, which showed even better hydrophobicity, with WCAs of 123 ± 0.6° and 157 ± 0.3°, respectively.

Organosilicon and fluorinated polymer are widely studied and broadly applied hydrophobic modification reagents in academia and engineering fields. They also show favorable performance advantages and application potential in the hydrophobic modification of CA membranes and other series of profiles. However, the modification of CA by organosilicon often must be completed through multiple chemical reactions, such as hydrolysis and condensation. The grafting rate and hydrophobic effect are influenced by multiple factors, such as the amount of hydroxyl (−OH) groups in CA molecules and the degree of reaction, which to some extent inhibits the development and application of this technology in the field of CA hydrophobic membranes. The accessibility and reactivity of fluorinated polymer with hydroxyl groups of CA are even weaker, which limits the hydrophobic modification degree. Moreover, the application of long-chain fluorinated polymers can cause the release of fluorinated pollutants. Therefore, the development of environmentally friendly, low fluorine content—and even fluorine-free—hydrophobic materials has become a new development direction.

#### 2.3.2. Other Hydrophobic Modifiers

In addition to the silicon/fluorine series hydrophobic modifiers, in recent years, some other types of hydrophobic groups/modifiers have been prepared for hydrophobic CA membranes through physical blending or chemical grafting, combined with the functional group properties and hydrophobic targets of CA polymers.

**Figure 5:**
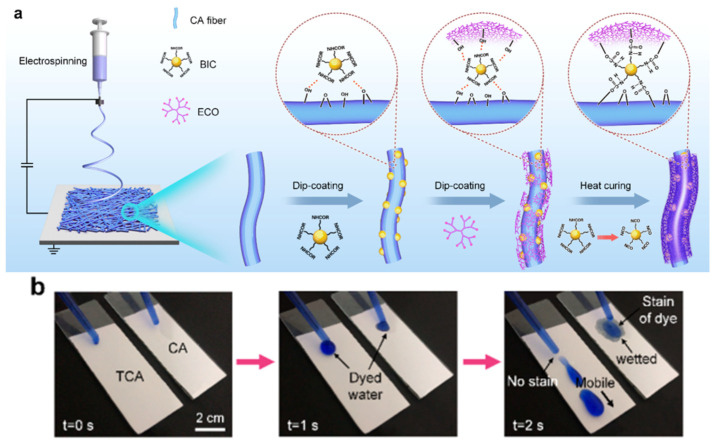
(**a**) Green synthesis route for high-performance waterproof and breathable membranes; (**b**) wettability of dye solution on CA and TCA tilted surfaces [54].

(1) Blending introduction is the introduction of hydrophobic groups/substances into profiles such as CA membranes through component blending, post-finishing, and composite methods based on van der Waals forces. Xiong et al. [49] blended CA with a styrene–butadiene–styrene block copolymer (SBS) and prepared asymmetric membranes by vapor-induced phase separation (VIPS), where the hydrophobic layer was composed of SBS and the WCA was greater than 145°. Barbar et al. [50] first used 1,2-epoxydodecane for the hydrophobic modification of methylcellulose, and then they mixed it with CA to prepare membrane materials by the NIPS method, with improved hydrophobicity. Ganesh et al. [51] prepared nanofiber membranes by blending thermally responsive poly(N-isopropyl acrylamide) (PNIPAM) with CA. Their results exhibited superhydrophobicity close to 0° at room temperature (23 °C) due to the inherent hydrophilicity of CA and PNIPAM as well as the intermolecular hydrogen bonds between them and water molecules. At a raised temperature (40 °C), PNIPAM underwent a phase transition, forming hydrogen bonds between CA and PNIPAM molecules, exposing the hydrophobic main chain, with a WCA greater than 130°. Zhang et al. [52] prepared a sandwich-structured Janus nanofiber membrane through layer-by-layer electrospinning, consisting of a hydrophobic PU layer (WCA = 123.8 ± 3°), a CA/PU transition layer (WCA = 88.7 ± 6°), and a hydrophilic CA layer (WCA = 64.3 ± 5°), with an oil contact angle (OCA) of 0°. Huang et al. [53] introduced poly(amideamine) (PAMAM) into the PLA/CA blend, and the presence of a large number of ester end groups in the PAMAM dendritic polymer improved the hydrophobicity of the blend, increasing the WCA from 66.3° to 79.9°. Blending introduction methods are simple and have the advantages of diverse material and process selections, but the bonding strength between hydrophobic components and CA substrates is the focus of this type of method.

(2) Grafting/cross-linking introduction is a chemical reaction that covalently bonds hydrophobic groups or compounds containing hydrophobic groups to CA molecular chains. Fluorine-free hyperbranched polymer materials can replace organic fluorine substances to achieve the hydrophobic modification of materials, effectively preventing the emission of fluorinated pollutants and achieving the goal of a sustainable reduction of environmental pollution. Professor Ding Bin’s team at Donghua University [54] modified CA nanofiber membranes using blocked isocyanates (BIC) and hyperbranched polymers (ECO) via a step-by-step dip-coating and heat-curing technology (Figure 5). Due to the bridging effect of BIC, CA and ECO were linked together through urethane bonds, forming a robust hierarchical structure within the membrane and greatly improving the adhesion and durability of the coating, ultimately achieving excellent hydrophobicity (WCA = 132.2°) and breathability (6.2 mm·s^−1^). Similarly, Zhang et al. [55] prepared emulsions for leather finishing through the reaction between -NH on the surface of SiO_2_ modified by KH550 and -NCO introduced on CA. The final film surface was smooth, dense, and hydrophobic (WCA = 104.3°). Su et al. [56] successfully synthesized a new carboxylate waterborne cellulose acetate (CWCA) emulsion with acrylic acid and hydroxyethyl acrylate as a modifier and isophorone diisocyanate as a bridging agent. The introduction of acrylate units led to the formation of a molecular mesh structure, limiting the water molecules from entering the membrane surface, with a WCA of 109.9°. Abdel Naby [57] modified CA with N-(aniline)maleimide and 4-nitro(aniline)maleimide, respectively, which can improve its hydrophobicity as well as its thermal and mechanical properties to a certain extent. Röhrl et al. [58] conducted thio-Michael click reactions between partial acrylate (acryl-substitution degree of 0.2) and various thiols. The introduction of fatty alkyl thiols increased the WCA of the material from 74° to over 100°. Xu et al. [59] used irradiation-induced and reversible addition–fragmentation chain transfer (RAFT) polymerization technology to achieve a controllable grafting of poly(glycidyl methacrylate) (PGMA) on the CTA membrane. When the PGMA content reached 31%, the water contact angle of the CTA membrane could reach 105.4°.

Different from microstructure modification, anchoring hydrophobic compounds or groups on the surface of CA membranes through chemical bonding is currently the mainstream technology for preparing hydrophobic membranes, endowing them with stable and persistent hydrophobic properties. However, there are some problems with this technology method in the modification of CA series membranes: (1) High acetylation CTA with less active group content makes it difficult to achieve in-depth modification for hydroxyl groups; (2) the modification process is prone to an uneven molecular weight distribution and side reactions, which can affect the reaction process and product purity; and (3) the possible slight reverse hydrolysis of hydroxyl groups during esterification can reduce the reaction efficiency between the catalyst and the product. Therefore, the selection of modifiers with high reactivity and excellent hydrophobic properties, as well as the design and establishment of mild reaction processes, remain significant research topics in CA membrane chemical hydrophobic modification. Table 1 summarizes the microstructure and chemical functions of the CA hydrophobic modification process characteristics.

## 3. Application of Hydrophobic Cellulose Acetate in Environmental Purification

Hydrophobic cellulose acetate is widely used in various fields due to its abundant raw material resources and unique performance properties. Especially in environmental purification such as water treatment, the biomass properties of cellulose acetate can effectively avoid secondary pollution, and its hydrophobic characteristics and film-forming properties enable it to selectively separate and purify different types of mixtures, demonstrating efficient and rapid separation efficiency.

### 3.1. Purification of Oil–Water System

With the continuous improvement of pollutant emission standards in the petrochemical industry, oleophilic hydrophobic separation membranes have attracted much attention in the scientific and engineering fields for their highly efficient and precise directional separation properties. Based on different processes that produce a variety of structural types of hydrophobic cellulose acetate membranes in the field of oil–water separation, research continues to deepen: phase separation can regulate the hydrophilic/hydrophobic properties and pore scale of the CA membrane surface; the CA nanofiber membrane has efficient wetting properties and strong capillary action due to its large specific surface area and high porosity, which can selectively adsorb or trap organic solvents in the aqueous phase, achieving the purpose of oil–water separation; and CA aerogel porous material has significant application potential in the purification of the oil–water system. The controllable separation of oil and water by the CA membrane with hydrophobic properties is mainly achieved by coupling the lipophilic properties of hydrophobic groups with the adsorption specificity of porous materials and the structural advantages of large specific surface area bonding sites.

#### 3.1.1. Phase Separation Porous Membrane

The phase-separation process is the most mature preparation technology for porous materials and membrane products. Zhang et al. [29] prepared hydrophobic porous CA profiles (WCA = 147°, oil contact angle 0°, porosity 92.1%) using the TINIPS method, which exhibited high selectivity and adsorption of various oil and organic solvents. More importantly, its hydrophobicity remained stable in harsh environments, such as pH = 1–14, temperature 0–70 °C, and high turbulence, demonstrating excellent weather resistance. The graphene/CA composite membrane prepared by Wang et al. [38] exhibited good hydrophobicity (151°) and saturated adsorption (6.71–14.72 times its self-weight) under a wide range of pH (1–14) and temperature (0–70 °C) conditions, with a separation rate of 99.1% for the oil phase component (*n*-hexane) from the aqueous phase. Wang et al. [60] prepared a zeolitic imidazolate frameworks-8 (ZIF-8) superhydrophobic CA foam material (ZIF-8/CA) using the TINIPS method, demonstrating a WCA value up to 153°. The addition of ZIF-8 transformed the microstructure of the foam material from a fiber-bundle structure to a coarse-skeleton porous structure. The composite membrane exhibited adsorption specificity and high efficiency for various oil agents, with a saturated adsorption capacity maintained at 6.89–14.61 g·g^−1^. The continuous oil–water separation flux and separation efficiency were 3250 L·m^−2^·h^−1^ and 98.7%, respectively. Liu et al. [30] obtained CTA hydrophobic membranes with high porosity (93.1%), large specific surface area (13.65 m^2^ g^−1^), and unique micro/nanostructures using the TIPS method, with a WCA value of 137.5°. Due to the efficient hydrophobic/lipophilic properties and strong capillary action, its oil adsorption rate reached 21.5 g·g^−1^, which could effectively purify oil–water mixtures.

#### 3.1.2. Nanofiber Membrane

Electrospinning technology can prepare CA fiber membranes with unique micro/nanofiber composite structures that have a high surface area/volume ratio, controllable pore size and membrane thickness, and high porosity and sufficient mechanical strength, as well as ultra-high flux. Based on these qualities, hydrophobic modification can maximize the oil–water separation efficiency, resulting in hydrophobic nanofiber membranes [46]. Shang et al. [61] coated fluorinated polybenzoxazine (F-PBZ)/SiO_2_ on the surface of CA nanofibers, with a maximum WCA of 161° and a minimum OCA of 3°. The membrane can achieve the rapid, efficient, and stable separation of oil–water mixtures, and it is suitable for the removal and treatment of oil stains. Arslan et al. [62] found that perfluorooctyltriethylsilane (FS) can cover the surface of CA nanofibers through hydrolysis and condensation reactions, making them superhydrophobic (155°). They can separate n-hexane/oil/water systems by simple extrusion adsorption, with an adsorption volume of up to three times their self-weight. Zhang et al. [52] prepared a PU-CA/PU-CA Janus membrane with a thickness of 80 μm via layer-by-layer electrospinning, with a permeability of 3.4 ± 0.4 × 10^4^ L·m^−2^ h^−1^ bar^−1^ and an oil (cyclohexane)–water separation efficiency of 99 ± 0.4%. Ma et al. [63] prepared a fluorinated polybenzoxazin (F-PB)/SiO_2_-modified polyamides (PI)/CA core–shell nanofibrous membrane, a self-supporting, highly flexible fibrous membrane with superhydrophobicity (162°), superhydrophilicity (approximately 0°), and super wettability. The membrane can effectively separate various oil–water mixtures solely by gravity, with characteristics such as high flux (3106.2 ± 100 L·m^−2^ h^−1^), high separation efficiency (>99.5%), and high separation stability (10 cycles, >98%). Xiong et al. [64] prepared a polystyrene/cellulose acetate/hydrophobic silica (PS/CA/HSiO_2_) composite membrane with excellent hydrophobic/oleophilic properties by optimizing the porosity and pore size distribution inside the fiber membrane, which exhibited excellent oil adsorbing and stability under extreme pH conditions. Yu et al. [65] prepared a superhydrophobic (WCA = 159.5°)/hydrophilic Janus membrane with unidirectional liquid-conducting properties by a plasma gas-phase graft polymerization of octamethylcyclotetrasiloxane (D4) on one side of a CA nanofiber membrane. The separation efficiency of various oil–water systems exceeded 98.7% and showed good reusability. Professor Ding Bin’s group had conducted a series of studies on such nanofiber materials, such as adding SiO_2_ [66], Al_2_O_3_ [67], and other nanoparticles, as well as F-PBZ [68], to improve the hydrophobicity of the nanomaterials and achieve efficient oil–water separation. Ding Siruo [69] used a combination of electrospinning and freeze-drying to prepare three-dimensional nanofiber sponges using CA and polyethylene oxide (PEO) as raw materials and modified them with methyltrichlorosilane (MTS) on the fiber surface to increase the WCA from 0° to 135°. The modified nanofiber sponges could selectively adsorb the oil in water and could be completely separated into oil and water by gravity alone. The adsorption capacity of silicone oil reached 130 times its self-weight. Zhang et al. [70] prepared environmentally friendly CA-based composite fiber mats with mechanically stable pore structures and coarse fibers. Based on phase migration during electrospinning, low surface tension thermoplastic polyurethane (TPU) flexible polymers tended to concentrate on the surface of CA/TPU composite fibers, resulting in hydrophobicity (>130°) and a high saturation adsorption capacity (28.35–64.18 g g^−1^). Weng et al. [71] combined electrospinning and surface silanization to form a superhydrophobic surface with a WCA of 153°, exhibiting self-cleaning and anti-fouling capabilities against dust and dirty water droplets. The oil–water separation efficiency exceeded 98.29% for liquids such as hexane, kerosene, and sunflower oil.

#### 3.1.3. Aerogels

An aerogel is a promising profile for mixture separation and environmental purification, with the advantages of being lightweight and demonstrating fast adsorption, large capacity, and reusability. Hydrophobic cellulose acetate aerogels combine the advantages of both raw materials and profiles and have attracted much attention for the separation and purification of oil phase components. Paulauskiene et al. [72] used the starch–CA mixture as the cross-linking agent of cellulose to synthesize hydrophobic aerogel, showing high hydrophobicity (WCA = 124~129°). Compared with the polyester resin aerogel, the adsorption capacity of the all bio-based aerogel was significantly higher, with 47 g·g^−1^ for crude oil, 51 g·g^−1^ for marine diesel oil, and 56 g·g^−1^ for lubricating oil, as well as good oil retention. However, the elasticity, stability, and recyclability of this aerogel needed to be further improved. Ruello and Kim [73] coated methyltrimethoxysilane (MTMS) on the surface of CA/PVA frozen gel to obtain hydrophobic gel materials (WCA = 118°~145°) with high saturated adsorption capacity and high separation flux. Wu et al. [74] used thermal cross-linking and silicone modification to prepare CA/PEO nanofiber aerogels with good hydrophobicity (WCA = 135.5°), excellent resilience (recovery after 50 compression cycles), high adsorption capacity (63~128 g·g^−1^), and reusable CA/PEO nanofiber aerogels. Tripathi et al. [75] further modified the CA aerogel with trichlorooctylsilane (TCOS), showing high hydrophobicity (WCA > 120°), low water adsorption (0.02 wt.%), and high oil adsorption (capable of adsorbing n-hexane more than 25 times its self-weight).

### 3.2. Separation and Purification of Saltwater Systems

Since the 1960s, Leob and Souririjan have been the first to produce cellulose acetate reverse osmosis membranes. Cellulose acetate has been used to make various types of separation membranes due to factors such as its abundant raw materials, good film-forming properties, high retention rate, environmental friendliness, and excellent chlorine resistance. It has been widely used in the fields of seawater desalination, brackish water desalination, and other saltwater system separations. However, these filtration membranes usually utilize the hydrophilic properties of cellulose acetate to function. With the continuous deepening of hydrophobic modification and application, hydrophobic-modified cellulose acetate series membranes have received special attention in the separation of saltwater systems or the removal of heavy metal salts.

Ferjani et al. [76] constructed poly(methylhydrosiloxane) (PMHS)-modified CA asymmetric composite membranes, which formed a hydrophobic top surface supported by a sponge-like structure. The PMHS coating also helped to enhance the dielectric repulsion and dehydration of ions during mass transfer through the active layer. The composite membrane enhanced both salt retention and water permeability compared to pure CA membranes. Using the gas phase-separation method, Laura et al. [77] prepared nanocomposite films containing propionylated lignin and CTA. Propionation could effectively improve the dispersion of lignin nanoparticles in the CTA matrix, promote changes in membrane roughness and wettability, and enhance the hydrophobicity of the films. Compared with pure CTA, all composite membranes carried electrical charges and facilitated the ionic removal by electrostatic action, achieving groundwater nanofiltration/reverse osmosis desalination and purification. Xu et al. [78] prepared a dense, ultrathin, and highly selective CTA/PVP hydrophobic functional layer (WCA of 100.5°) on PAN nanofiber membranes using vertical dissolution. The optimal process resulted in a separation membrane with a retention rate of 86.8% for divalent salt ions (MgSO_4_ aqueous solution) and a water flux of 58.6 L m^−2^ h^−1^, exhibiting typical nanofiltration separation characteristics. Iqhrammullah et al. [79] prepared CA-PU films by a polycondensation reaction between CA and diphenylmethane diisocyanate (MDI). The wettability of the film tended to be hydrophobic after adding PU, and nitrogen-containing functional groups (NH and NCO) could show a high purification capacity of adsorption of high-toxicity salt ions (such as Pb^2+^) in aqueous solutions of heavy metal salts through chelation.

The most widely used hydrophobic CA membrane in saltwater systems is based on using pervaporation (or membrane distillation) to achieve the separation and purification of high-concentration saltwater. Traditional membrane water-treatment technology has the disadvantages of high energy consumption, cumbersome operation processes, and high influent concentration [80]. Membrane distillation technology, due to its unique advantages, has shown great potential in energy conservation, emission reduction, and environmental protection [81,82]. However, there are still many challenges in using polymer membranes for MD. The use of biodegradable polymers in MD can help reduce the use of non-biodegradable petroleum-based polymer membranes [83]. Hydrophobicity being a fundamental requirement for MD in most applications, surface modification of the developed membrane is necessary to design a dual-layer membrane with both hydrophobic and hydrophilic layers [84]. Sayed et al. [85] prepared a PCL/CA dual-layer hydrophobic/hydrophilic membrane by electrospinning. SiO_2_ was electrosprayed onto the top layer of the PCL/CA membrane. The membrane surface structure was transformed from a nanofiber surface to a particle surface microstructure. The hydrophobicity increased from 119.0° to 152.4°, and the direct membrane distillation (DCMD) flux remained stable at 15.6 kg m^−2^ h^−1^, significantly increasing the desalination rate to 99.97%. Ioannou et al. [86] demonstrated the persistent anti-fouling performance of superhydrophobic membranes treated with plasma O_2_ etching and C_4_F_8_ deposition in air-gap membrane distillation (AGMD) and DCMD. Among them, the PTFE membrane was treated only on the top layer, and the CA membrane was treated on both sides, with the water static contact angle above 160° and contact angle hysteresis around 10°. Due to the surface alternation effect, the percentage increase in porosity and the average pore size of the plasma-treated CA membrane was greater in range compared with the PTFE membrane. Therefore, the plasma-treated CA membrane had a higher flux value of 3.9 L m^−2^ h^−1^ in AGMD and 9.3 L m^−2^ h^−1^ in DCMD. Eljaddi et al. [87] developed a specific membrane for photovoltaic seawater desalination using theoretical and experimental methods, with PVDF as the porous carrier (126°) and CTA as the thin and dense coating layer (57°). The composite membrane exhibited good compatibility between the CTA polymer and the PVDF carrier, and CTA did not intrude into the porous hydrophobic materials. Under the same conditions, the composite membrane had a stable desalination performance when tested with pure water, saltwater, and even surfactants (SDS). Compared with the original PVDF membrane (a fully hydrophobic membrane), no wetting occurred, and a high desalination rate (close to 100%) was achieved.

### 3.3. Water-Repellent Protection

With the expansion of global economic activity, icing, as a regular natural phenomenon in daily life, seriously threatens the production and life of human beings [88,89,90]. It is urgent to solve the problem of icing. Traditional mechanical or chemical methods have the disadvantages of high pollution, high energy consumption, and low efficiency, making solar energy an ideal energy source for anti/de-icing processes [91,92]. The key to the anti-icing and anti-fouling technology of superhydrophobic coatings is to reduce the adhesion between the ice and the substrate, that is, to improve the hydrophobicity of the surface. Tan et al. [93] combined porous cellulose acetate membranes (substrate) with photothermal conversion material carbon nanotubes (photothermal layer) and solid paraffin (lubricant) to prepare a photothermal solid smooth surface with excellent overall performance (WCA > 150°). On the other hand, radiation cooling technology that can achieve thermal management without energy has shown great potential in energy conservation, emission reduction, and environmental protection [94,95]. However, hydrophilic materials are prone to wetting, resulting in poor surface anti-pollution capabilities. Thus, it was essential to improve the hydrophobicity of thin membrane materials to provide non-wetting and self-cleaning capabilities and achieve sustainable radiation cooling performance. The inherent molecular vibrations endowed the CA membranes with high mid-infrared emissivity. Li et al. [96] manufactured a layered CA membrane using expandable multi-needle electrospinning with high mid-infrared emissivity as well as solar reflectivity due to the porous structure. Zhang et al. [97] added hydrophobic SiO_2_ particles in the CA membrane to endow it with superhydrophobicity. Liu et al. [98] designed a silicon hybrid cellulose acetate (SHCA) aerogel cooler with a porous microstructure by combining phase-separation and freeze-drying methods. The contact angle was increased to 150.2°, and its solar reflectivity and atmospheric transparency window (ATW) emissivity reached 96 and 97%, respectively. Overall, superhydrophobic anti-icing technology and radiative cooling technology serve different application fields, but both are based on in-depth research and innovative applications of material surface properties. The development of these two technologies not only helps solve specific engineering problems but also promotes progress and innovation in the field of materials science.

Hydrophobic cellulose acetate membranes have great application value in water-repellent protective packaging materials. A green packaging design first needs to consider the recyclability and biodegradability of materials, thereby reducing the risk of groundwater pollution caused by landfill leachate [99]. CA membranes with biomass properties can be used as raw materials for packaging preparation, but due to high hydrophilicity and poor moisture resistance [100,101], they must be modified to meet the requirements of food packaging. Jiang et al. [102] designed a multifunctional black film with a sandwich structure (filter paper/iron chelated sodium lignosulfonate/cellulose acetate, P/SLS-Fe_4_/CA) in which the CA solution was coated on both sides of P/SLS-Fe_4_ using the casting method, and CA formed a dense structure to fill the pores of the filter paper. The dynamic reversible physical bonding between the three materials endowed the composite material with enhanced mechanical properties, and the WCA increased from 58.4° to 138.7°. Dong et al. [103] successfully prepared a homogeneous and highly permeable pine-derived cellulose acetate (PDCA)-based nanocomposite film in which the interaction between the nano-SiO_2_ and cellulose acetate molecular chains was through the hydrogen bond formed between the hydroxyl groups. This resulted in effective improvement of the barrier, hydrophobic (from 59.18° to 67.82°), and mechanical properties of the cellulose acetate composite film. Shorey et al. [104] found that samples loaded with oleic acid-esterified lignin had a higher contact angle value (about 90°, increased by 37.9%), better mechanical properties, and stronger UV-blocking properties. Singh et al. [105] dissolved CA and esterified hydroxyethyl lignin together and coated the paper, increasing the contact angle from 52.4° to 133.7°.

### 3.4. Other Systems

The residues of drugs, dyes, and other substances in industrial wastewater pose great harm to the environment and ecosystem [106]. Hydrophobic cellulose acetate membrane materials also have great potential for the directional catalytic degradation of organic matter. Tian et al. [107] developed a modified CA-based electrospinning nanofiber aerogel (T-ENA) for the treatment of oil and drug residues in complex wastewater and water remediation. Due to silane cross-linking, T-ENA exhibited enhanced mechanical stability/resilience and hydrophobicity (WCA = 143.5°) without affecting its high porosity (>98%) or low density (10 mg cm^−3^). The results indicated that hydrophobic T-ENA had a separation efficiency of over 99% for different oil–water solutions. Meanwhile, the residue of carbamazepine (CBZ) in wastewater could be rapidly degraded within 20 min to the enhanced adsorption catalytic ability and activation of peroxomonosulfate (PMS) by the loaded ZIF-67. Hydrophobic-modified cellulose acetate and its series of membrane products have also demonstrated excellent separation and purification properties in many other fields. Penabad-Peña et al. [108] added the block copolymer poly-4-vinylpyridine-b-epoxyethane (P4VP-b-PEO) into the CA casting solution and formed the membrane by the NIPS method. P4VP-b-PEO promoted the existence of hydrophobic air molecules in the pores, thereby reducing the penetration of water molecules into the membrane. The porous structure increased the specific surface area of the membrane and enhanced the π-π interaction between the block copolymer and electron-deficient pollutants. This resulted in stronger electrostatic interactions between the membrane and the contaminant, which substantially enhances the adsorption capacity of the composite membrane, thus providing a novel concept for the targeted removal of specific electron-deficient contaminants (mainly pharmaceuticals) from water. Kosaka et al. [109] improved the targeted adsorption of lipase-specific substrates by annealing CA ultrathin films to reduce the wettability (hydrophobicity enhancement), thus providing insights into the development of carriers or protein-separation membranes for biomolecules with specific binding properties. Abad et al. [110] constructed a CA/Au/ZnO nanocomposite material with photocatalytic activity for the degradation of biological dyes, and the addition of nanoparticles increased the WCA by about 20%. It also enhanced the photoreactive sites and improved the photocatalytic performance. Kwon et al. [111] prepared TiO_2_-CA nanofibrous membranes using emulsion electrospinning. The hydrophobicity was increased from 112.5° to 140° due to the micro-folded and raised surface morphology of the nanofibers. Excellent filtration efficiency (99.6%) was shown for particulate matter (PMs), and the material showed a photocatalytic removal efficiency of 78.6% for NO.

Hydrophobic-modified cellulose acetate and its series of membrane products have also demonstrated excellent separation and purification properties in many other fields. Hydrophobic methylcellulose/CA hybrid membranes prepared by Barbar et al. [50] achieved partial denitrification of emulsions. Joseba et al. [112] prepared CA-based mats modified by TiO_2_ nanoparticles synthesized from poly(ethylene oxide-propylene oxide-ethylene oxide) triblock copolymer and sol–gel by electrospinning technology. The lower fiber diameter and pore diameter, as well as the higher fiber density, resulted in high hydrophobicity (WCA is 140° ± 6°) and self-cleaning ability. Kaschuk et al. [113] used pyromellitic dianhydride (PMDA) as a cross-linking agent and triethylamine (TEA) as a catalyst to cross-link tetraethyl orthosilane (TEOS) and octyl trichlorosilane (OCTS) via the sol–gel method for the hydrophobic modification, increasing the contact angle (from 56° to 91°) and the solvent resistivity. Cao et al. [114] prepared cellulose membranes (CMs) and fluorinated cellulose membranes (FCMs) with different functional groups and hydrophobic properties using CA as a raw material. These were respectively used as friction-positive and friction-negative layers for FCM/CM-based friction nanogenerators (FCTENG), with a contact angle of up to 140°. Mogharbel et al. [115] incorporated rare earth-activated strontium aluminium oxide (RSAO) nanoparticles into the nanofibers of cellulose acetate–polycaprolactone (CA-PCL) composites, increasing hydrophobicity from 130.6° to 150.7°. Tian et al. [116] developed a Janus fiber membrane composed of hydrophobic gauze modified with 3-(trimethoxysilylpropyl) methacrylate (TMSPMA) and hydrophilic electrospinning cellulose acetate fiber membrane doped with carbon nanotubes (CNT). Utilizing its asymmetric wettability along the thickness direction, it acted as a “liquid diode” to achieve heat-collection performance and unidirectional liquid transport.

## 4. Conclusions

In summary, cellulose acetate and its series of membrane materials can obtain excellent lipophilicity and other properties after hydrophobic modification treatment, which changes some of the limitations of the original hydrophilic properties of the materials and better expands their applications in the field of membrane separation. The directional design and construction of nano/microstructures can enhance the hydrophobic properties of membrane materials by capturing air molecules and reducing the contact area to increase the energy barrier for water-molecule penetration. Chemical modification of the introduction of hydrophobic groups or low surface energy components can be made directly on the surface of the membrane material to form a hydrophobic barrier. The two strategies can be used alone or coupled to achieve a targeted hydrophobic modification of cellulose acetate and its series of membrane products, and the resulting membrane products in the separation, purification, decontamination, and other fields play important roles. Although the related research and application have made significant breakthroughs and stage-by-stage progress, there are still many deficiencies in the depth of research and the development and breadth of application. In the future, the study of hydrophobic cellulose acetate and its series of membranes needs to focus on the following aspects: (1) the uniformity and stability of hydrophobic nanostructures during the construction process; (2) the secondary contamination of the chemical hydrophobic modification process; and (3) the balance between hydrophobic modification and membrane-separation efficiency. On this basis, the hydrophobic modification of cellulose acetate can be combined or synchronized with other functional modifications to expand its application fields. For example, inorganic particles with photocatalytic properties can be selected as modification additives. By controlling the dispersion of inorganic particles on the membrane surface, a rough structure can be constructed to prepare multifunctional composite membranes with both hydrophobic properties and photocatalytic activity. By selecting functional substances with both hydrophobic and antibacterial properties, cellulose acetate membranes can be modified simultaneously to develop multifunctional products for the medical and healthcare fields (e.g., infant and toddler hygiene products).

## Figures and Tables

**Table 1:** Different hydrophobic modification processes of cellulose acetate.

Modification Type	Polymer	Specific Process	Key Substance	WCA	Reference
Improved molding process	CA	TINIPS	-	147°	[29]
	CTA	TIPS	-	137.5°	[30]
	CA-PAN	Electrospinning	-	131°	[31]
	CTA	Electrospinning	-	141.6°	[32]
	CA	Template	-	130–140°	[33,34]
Introduction of exogenous substances	CA	Electrospinning	SiO_2_	108°	[35]
	CA	Electrospinning	ZnO	124°	[36]
	CA-CA/ZnO	Pair spray	ZnO	152°	[37]
	CA	TINIPS	Graphene	151°	[38]
Post-treatment	CA	Electrostatic interactions	SiO_2_	145 ± 0.08°	[39]
	CA	Coating	SiO_2_	154°	[40]
	CTA	Respiration chart	-	120 ± 1.3°	[41]
Organic silicon/fluorine modifiers	CA	Cross-linking	Siloxane	>100°	[44]
	CA	Coating	PDMS/SiO_2_	157.3 ± 3°	[46]
	CA/SiO_2_	Coating	FAS	156°	[47]
	CAAs	Chemical vapor-phase	PFDA	136°	[48]
Blending introduction	CA	VIPS	SBS	145°	[49]
	CA	Blending	PNIPAM	>130°	[51]
	PU-CA/PU-CA	Layer-by-layer electrospinning	PU	123.8 ± 3°	[52]
	PLA/CA	Blending	PAMAM	79.9°	[53]
Grafting/cross-linking introduction	CA	Grafting	BIC	132.2°	[54]
	CA	Grafting	SiO_2_	104.3°	[55]
	CA	Cross-linking	Acrylic	109.9°	[56]
	CTA	Thio-Michael click reactions	Fatty alkyl thiols	100°	[58]
	CTA	RAFT	PGMA	105.4°	[59]

## Data Availability

No new data were created or analyzed in this study. Data sharing is not applicable to this article.
